# High-throughput epitope profiling of antibodies in the plasma of Alzheimer’s disease patients using random peptide microarrays

**DOI:** 10.1038/s41598-019-40976-x

**Published:** 2019-03-14

**Authors:** Kyu-Young Sim, Sang-Heon Park, Kyu Yeong Choi, Jung Eun Park, Jung Sup Lee, Byeong C. Kim, Jeonghwan Gwak, Woo Keun Song, Kun Ho Lee, Sung-Gyoo Park

**Affiliations:** 1 School of Life Sciences, Gwangju Institute of Science and Technology (GIST), Gwangju, Republic of Korea; 2 National Research Center for Dementia, Chosun University, Gwangju, Republic of Korea; 3 Department of Biomedical Science and BK21-plus Research Team for Bioactive Control Technology, Chosun University, Gwangju, Republic of Korea; 4 Department of Neurology, Chonnam National University Medical School, Gwangju, Republic of Korea; 5 Biomedical Research Institute & Department of Radiology, Seoul National University Hospital, Seoul, Republic of Korea

## Abstract

The symptoms of Alzheimer’s disease (AD), a major cause of dementia in older adults, are linked directly with neuronal cell death, which is thought to be due to aberrant neuronal inflammation. Autoantibodies formed during neuronal inflammation show excellent stability in blood; therefore, they may be convenient blood-based diagnostic markers of AD. Here, we performed microarray analysis of 29,240 unbiased random peptides to be used for comprehensive screening of AD-specific IgG and IgM antibodies in the blood. The results showed that (1) sequence-specific and isotype-specific antibodies are regulated differentially in AD, and combinations of these antibodies showing high area under the receiver operating characteristic curve values (0.862–0.961) can be used to classify AD, (2) AD-specific IgG antibodies arise from IgM antibody-secreting cells that existed before disease onset and (3) target protein profiling of the antibodies identified some AD-related proteins, some of which are involved in AD-related signalling pathways. Therefore, we propose that these epitopes may facilitate the development of biomarkers for AD diagnosis and form the basis for a mechanistic study related to AD progression.

## Introduction

Alzheimer’s disease (AD) is the most common cause of dementia, accounting for 60–80% of cases; signs include memory loss, problems with speaking or writing, and changes in personality^1^. The major markers of AD include deposition of aggregated Aβ with plaque development, and hyperphosphorylated tau with tangle formation, both of which are accompanied by neuronal damage and death^2^. In addition, AD places caregivers under considerable emotional, physical and economic pressure and imposes a great financial cost on the medical system; therefore, many studies have been undertaken in an attempt to find an effective treatment. However, AD remains incurable, and there is much controversy with respect to drug treatment^3^. As a result, it is important to prevent AD through a convenient diagnosis system.

The most well-established clinical method for diagnosing AD is detection of total tau, phosphorylated tau and β-amyloid (Aβ) 42 by conducting cerebrospinal fluid (CSF) analysis and positron emission tomography (PET) imaging^4^. However, these methods have some limitations. In the case of CSF analysis, differences between experimental results are problematic. Therefore, much effort has been made to develop international standards for diagnoses based on CSF analysis^5^. Another problem with CSF analysis is the high cost of testing, lack of availability and associated complications such as back pain, headache and increased intracranial pressure^4^. With respect to diagnosis based on PET using tracers, problems include initial costs, high ongoing costs and inaccuracies due to misinterpretation by the radiologist^6,7^. Therefore, many studies aim to develop new plasma biomarkers such as microRNA, cytokines and autoantibodies that can overcome the limitations associated with AD diagnosis^8^.

Antibodies are a vital part of the adaptive immune system. However, some antibodies, called natural autoantibodies, recognize self-antigens. These autoantibodies include primarily low affinity IgM isotypes produced spontaneously in healthy individuals during the process of B-1 cell development, and high affinity IgG isotypes generated through a process called affinity maturation^9–11^. Studies of autoantibodies in AD show that IgG-positive neurons are abundant in AD brains and that brain-reactive autoantibodies are present in the sera of AD patients^12^. In addition, a previous study identified AD-related autoantibodies targeting Aβ, tau protein and glia markers^9^. Another study based on a protein microarray consisting of 9,486 human protein antigens suggests that plasma IgG may be a diagnostic biomarker for AD^13^. Thus, studies of antibodies from AD patients show an association between AD pathology and the humoral immune system. However, these studies focused on the antibody-mediated recognition of whole proteins. Recent studies attempted to identify disease-specific antibodies in order to increase the disease specificity of biomarkers; these studies screened antibodies targeting peptide epitopes rather than proteins^14,15^. The data suggest that more effective biomarkers, which cannot be detected by screening for whole proteins, may be identified by screening for peptide epitopes. Other related studies have been undertaken in the AD field. One study used high-throughput screening of 4,608 octameric peptoids to identify AD-specific IgG antibodies. The study used an unbiased approach based on peptoids. However, a peptoid library cannot possibly mimic native peptide antigens presented to the immune system *in vivo*^16^. Thus, the use of peptoids has disadvantages with respect to target identification. Another study used a microarray comprising 10,000 random-sequence 20-mer peptides. However, this analysis did not include results for a set of target protein properties and only provides IgG class antibody profiles against only 10,000 peptide probes relevant to AD^17,18^.

Here, we used a high-throughput screening technique based on a random peptide microarray to screen for antibodies targeting random peptides. This technique has been used in several studies to examine humoral immune responses associated with development of pathological processes related to specific diseases, and for identification of new biomarkers for cancer, myalgic encephalomyelitis and valley fever^19–21^. We identified up- and downregulation of IgG and IgM antibodies in AD plasma that target specific peptide epitopes. Furthermore, we identified several interesting features of these antibodies. Differentially regulated IgG antibodies were derived from pre-existing IgM-secreting cells, likely through class switching. Several of the target proteins identified based on selected epitopes are known to be associated with AD. In addition, we found that the antibody profiles can be used to identify AD-related pathways. Thus, we have identified peptide biomarkers that may be useful as biomarkers for AD diagnosis and provide clues to the mechanisms underlying AD pathology.

## Results

### Identification of differentially regulated antibodies in AD

**Figure 1:**
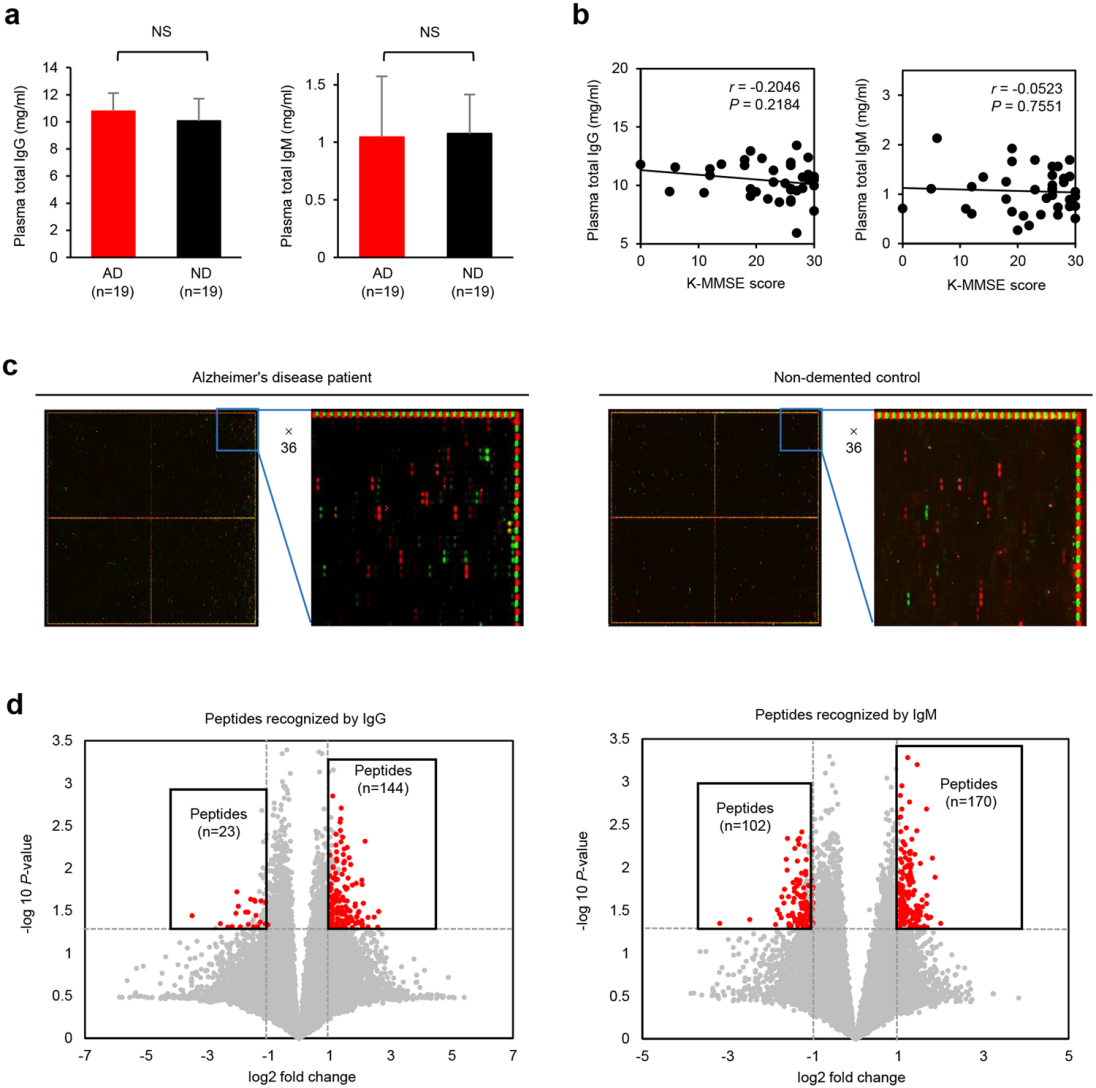
Identification of antibodies differentially expressed in Alzheimer's disease (AD). (**a**) Concentration of total plasma IgG and IgM in AD and ND subjects, as measured by enzyme-linked immunosorbent assay. (**b**) Correlation between total plasma IgG and IgM levels and the K-MMSE score (determined by calculating Pearson’s correlation coefficient). (**c**) Representative image of a scanned random peptide microarray for test subjects. The points in the scanned image represent anti-IgG (red) and anti-IgM (green) antibodies (middle), and anti-HA (red) and anti-FLAG (green) antibodies (edge). (**d**) Volcano plots show peptides (red points) recognized by IgG and IgM antibodies differentially expressed in AD (*P* ≤ 0.05, -fold change ≥2). Results are expressed as the mean ± SD. *P ≤ 0.05; **P ≤ 0.01; and ***P ≤ 0.001 (Student’s t-test) (**a**). *r* and *P* represent Pearson’s correlation coefficient and its associated *P*-value, respectively (**b**).

To screen for antibodies specifically regulated in AD, we used age-matched samples from Korean women: samples were obtained from AD patients (n = 19) and non-demented (ND) individuals (n = 19). Diagnoses were validated by magnetic resonance imaging or amyloid PET images and by Korean Mini-Mental State Examination (K-MMSE) scores (Table 1). All of the test subjects were female to reduce potential gender variation; this is because AD is more prevalent in females^22^. These subjects show high genetic frequency of the APOE ε4 genotype: the percentage of AD subjects with the ε3/4 genotype was 42.1%, whereas that of ND subjects was 10.5%. Conversely, APOE genotypes containing the ε2 allele were less frequent in AD: 5.3% of AD subjects had the ε2/3 genotype compared with 15.8% of ND subjects (Table 1)^23^. Before screening for antibodies targeting specific peptides, we analysed plasma total IgG and IgM levels to exclude the possibility that the results may be due to simple changes in total antibody levels. There was no significant difference between AD and ND subjects in terms of plasma total IgG and IgM concentrations; in addition, the concentrations did not correlate with K-MMSE scores (Fig. 1a,b). Random peptide arrays were printed with 29,240 random 15-mer peptides, which were then used to screen for differentially regulated antibodies in plasma from AD and ND subjects. Both IgM and IgG isotypes targeting specific peptides were detected using different fluorochrome-tagged secondary antibodies (Fig. 1c). Raw fluorescence intensities were obtained and then normalized using a quantile method. Further analysis involved pre-selection based on -fold changes in the signal between AD and ND samples and the calculated *P*-value. The results identified upregulated IgG (n = 144) and IgM (n = 170), and downregulated IgG (n = 23) and IgM (n = 102), antibodies in AD sera compared with ND sera (Fig. 1d). These data indicate AD-specific regulation of antibodies recognizing certain peptide epitopes; upregulation of IgM was observed most frequently, followed by upregulation of IgG.

**Table 1:** Subject characteristics.

Clinical diagnosis	AD (n = 19)	ND (n = 19)	*P*-value
Age (mean ± SD)	74.1 ± 7.3	73.0 ± 4.4	0.590^*^
K-MMSE score (mean ± SD)	17.7 ± 7.0	27.5 ± 1.6	<0.0001^*^
Sex (% female)	100	100	1*
Total IgG (mg/ml ± SD)	10.8 ± 1.4	10.1 ± 1.6	0.161^*^
Total IgM (mg/ml ± SD)	1.04 ± 0.5	1.08 ± 0.3	0.838^*^
IL-12p70 (pg/ml ± SD)	0 ± 0	0.34 ± 1.50	0.331^*^
TNF (pg/ml ± SD)	0.11 ± 0.3	0.24 ± 0.5	0.362^*^
IL-10 (pg/ml ± SD)	0.48 ± 2.1	0.09 ± 0.4	0.438^*^
IL-6 (pg/ml ± SD)	2.41 ± 2.8	0.74 ± 0.8	0.020^*^
IL-1β (pg/ml ± SD)	0 ± 0	0.11 ± 0.5	0.331^*^
IL-8 (pg/ml ± SD)	5.65 ± 3.9	4.21 ± 2.5	0.191^*^
*APOE* genotype, (%)			
ε2/3	5.3	15.8	0.290^†^
ε3/3	47.4	73.7	0.097^†^
ε3/4	42.1	10.5	0.027^†^
ε2/4	5.3	0	0.311^†^

Data are expressed as the mean (standard deviation) or as a percentage. Statistical significance was determined using Student’s t-test^*^ or the Chi-square test^†^. A *P*-value ≤ 0.05 was considered significant.

Abbreviations: AD, Alzheimer’s disease; ND, non-demented; SD, standard deviation; K-MMSE, Korean Mini-Mental State Examination; *APOE*, apolipoprotein E.

### Potential diagnostic utility of selected peptides

**Figure 2:**
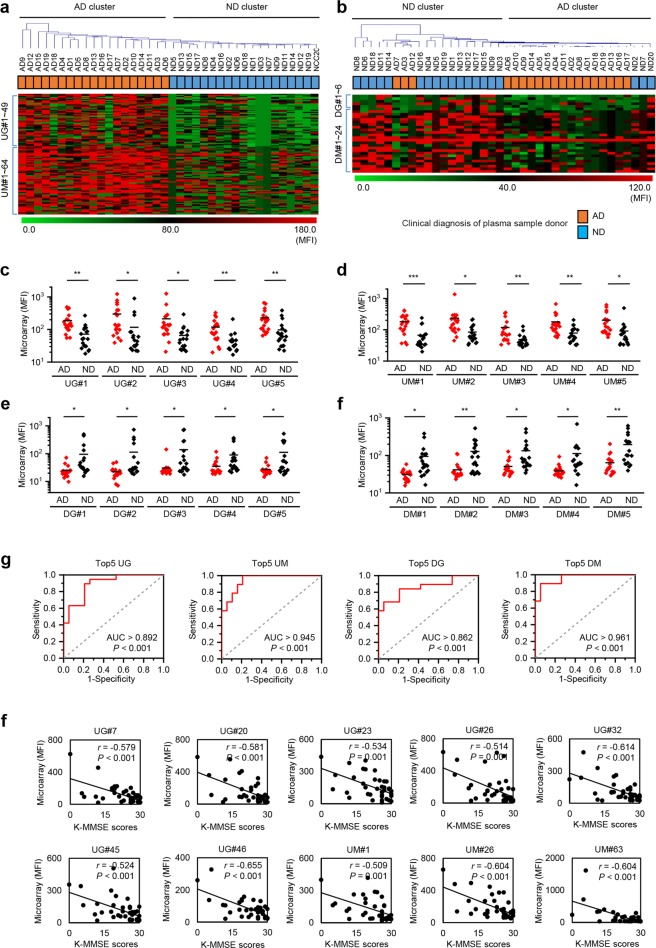
Assessment of diagnostic utility of peptides recognized by antibodies differentially expressed in Alzheimer’s disease (AD). (**a**,**b**) Hierarchical clustering analysis combined with a heatmap showing peptides (n = 113) recognized by upregulated IgG and IgM antibodies in AD (**a**) and peptides (n = 30) recognized by downregulated IgG and IgM in AD (**b**). (**c**–**f**) Dot plots for the top five peptides with a high area under the receiver operating characteristic curve value recognized by upregulated IgG (**c**) and IgM (**d**) and by downregulated IgG (**e**) and IgM (**f**), antibodies. *P ≤ 0.05; **P ≤ 0.01; and ***P ≤ 0.001 (Student’s t-test). Assessment of diagnostic utility of combinations with top five peptides targeted by upregulated IgG and IgM, and downregulated IgG and IgM, in AD (**g**). Correlation analysis between K-MMSE scores and peptides (*P* ≤ 0.001) recognized by antibodies differentially expressed in AD (**f**). *r* and *P* represent Pearson’s correlation coefficient and its associated *P*-value, respectively. For peptide ID: UG and UM indicate peptides recognized by upregulated IgG and IgM, respectively, and DG and DM indicate peptides recognized by downregulated IgG and IgM, respectively.

An area under the receiver operating characteristic curve (AUC) value of ≥0.75 was used as the cut-off value for selecting useful diagnostic peptides. Thus, in AD sera we identified 49 peptides targeted by upregulated IgG, 64 by upregulated IgM, 6 by downregulated IgG and 24 by downregulated IgM; these were selected for further analysis (Supplementary Figs S1 and S2; Table S1). Hierarchical clustering analysis of the selected peptides revealed that upregulated IgM and IgG antibodies clearly divided subjects into AD and ND clusters, while downregulated IgM and IgG antibodies did not (Fig. 2a,b). To improve the accuracy of diagnosis, we combined the fluorescence intensity readings of the top five peptides (Fig. 2c–f). This combination of peptides showed high AUC values for upregulated IgM (0.945), upregulated IgG (0.892), downregulated IgG (0.862) and downregulated IgM (0.961) (Fig. 2g). In addition, further analyses revealed that only ten peptides recognized by antibodies upregulated in AD correlated significantly with the K-MMSE score (which is a measure of cognitive impairment) (*P* ≤ 0.001, r < −0.5) (Fig. 2f; Supplementary Table S1). These results suggest that the selected peptides can be used as a biomarkers of AD progression.

**Figure 3:**
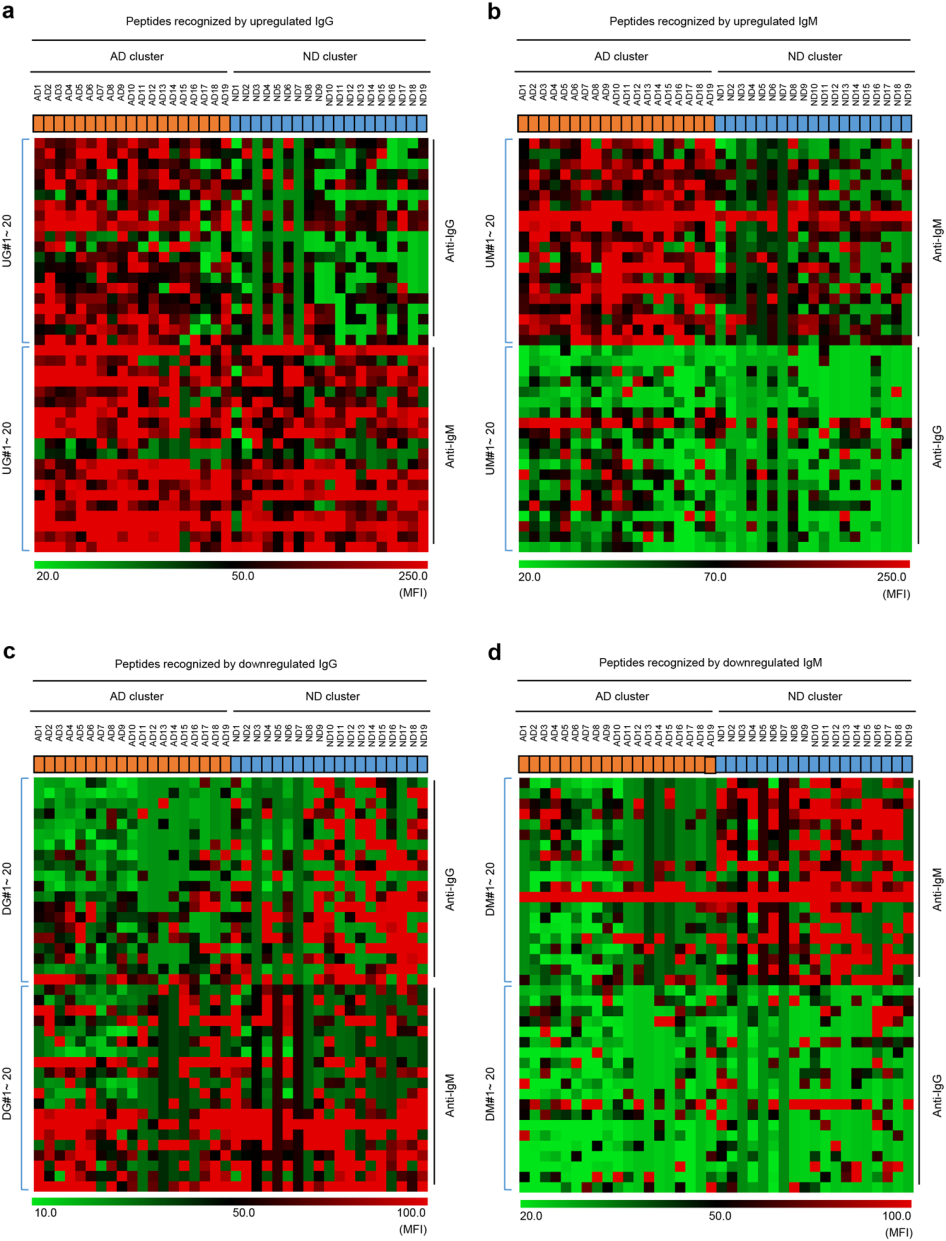
Identification of isotype changes in antibodies differentially expressed in Alzheimer’s disease (AD). (**a**–**d**) Heatmap analysis of the top 20 peptides targeted by upregulated IgG (**a**) and IgM (**b**) antibodies, and by downregulated IgG (**c**) and IgM (**d**) antibodies, in AD (upper). The underlying heatmap shows the corresponding IgM (**a**,**c**) and IgG (**b**,**d**) that recognize the same peptides. For peptide ID: UG and UM indicate peptides recognized by upregulated IgG and IgM, respectively, and DG and DM indicate peptides recognized by downregulated IgG and IgM, respectively.

To observe differences in the isotype of antibodies binding to the selected peptides, we performed heat map analysis of the top 20 peptides recognized by IgM and IgG isotypes (Fig. 3a–d). The results revealed that AD-specific IgG antibodies (the levels of pre-existing IgM antibodies for the same peptides were already high in both AD and ND sera) were derived from pre-existing IgM-secreting cells (Fig. 3a), whereas AD-specific IgM antibodies (the levels of IgG antibodies for the same peptides were low in both AD and ND sera) were newly generated (Fig. 3b).

### Identification of AD-related signal pathways

Next, the protein Basic Local Alignment Search Tool (BLAST) was used to identify potential target proteins containing the selected peptides. Before protein BLAST analysis, we predicted the epitopes likely exposed on such peptides to reduce the number of target protein candidates identified after input of random amino acid sequences. The predicted epitope sites were analysed using BepiPred-2.0 (threshold = 0.4), which can predict B cell epitope regions based on a random forest approach^24^. The epitopes were then analysed by the National Center for Biotechnology Information (NCBI) BLAST program using the UniProtKB/Swiss-Prot (Swiss-Prot) database and the search term “organism humans” (taxid: 9606); the most probable potential antigenic proteins for each epitope were selected (Table 2; Supplementary Table S2). Because we did not detect changes in the levels of inflammatory cytokines in the plasma of AD patients (an exception was IL-6), we only searched against “organism human” to reduce the number of possible target organisms. A previous report shows that plasma levels of IL-6, IL-1β and TNF-α in AD patients increased significantly^25^; however, we detected an increase only in IL-6 (Table 1).

**Table 2:** Representative for identified potential antigenic proteins for antibodies regulated in AD.

Peptide ID	Predicted epitope	Subject	Identity	Protein name	Sequence ID	Relativeness with AD
UG#5	IRLKEFTDYL	KEFTDYL	7	SOS1	Q07889.1	Increased in pyramidal neurons of AD patients
UG#23	QPIFDWYVP	QPIFD	5	TNFRSF21	Q75509.1	Trigger neuronal death through APP binding
UG#32	VQWDQCTIW	QWD + CT	5.5	ATM	Q13315.4	Interact with ATBF1 mediated neuronal death by Aβ_42_
UG#37	DNFFWEVWV	+NFFWE	5.5	S100A1	P23297.2	Knockout decreases plaque number and load
UM#57	IRLKEFTDYL	KEFTDYL	7	SOS1	Q07889.1	Increased in neuronal cells of AD patients
UM#59	CKRFDCPTPI	KRF + CP	5.5	SP4	Q02446.2	Increased in brain of AD patients, associated with NFT and neuronal apoptosis
DM#21	IYSCFRWKIF	+YSCFR	5.5	GNPAT	O15228.1	Decreased in AD mouse and human brain
DM#23	QFQFPWMNY	PWM + Y	4.5	CNTN2	Q02246.1	Decreased in brain of AD patients, correlated with BACE1

Abbreviations: AD, Alzheimer’s disease; APP, amyloid precursor protein; Aβ_,_ β-amyloid; NFT, neurofibrillary tangle. As peptide ID, UG and UM indicate peptides for upregulated IgG and IgM, DM indicates peptides for downregulated IgM.

**Figure 4:**
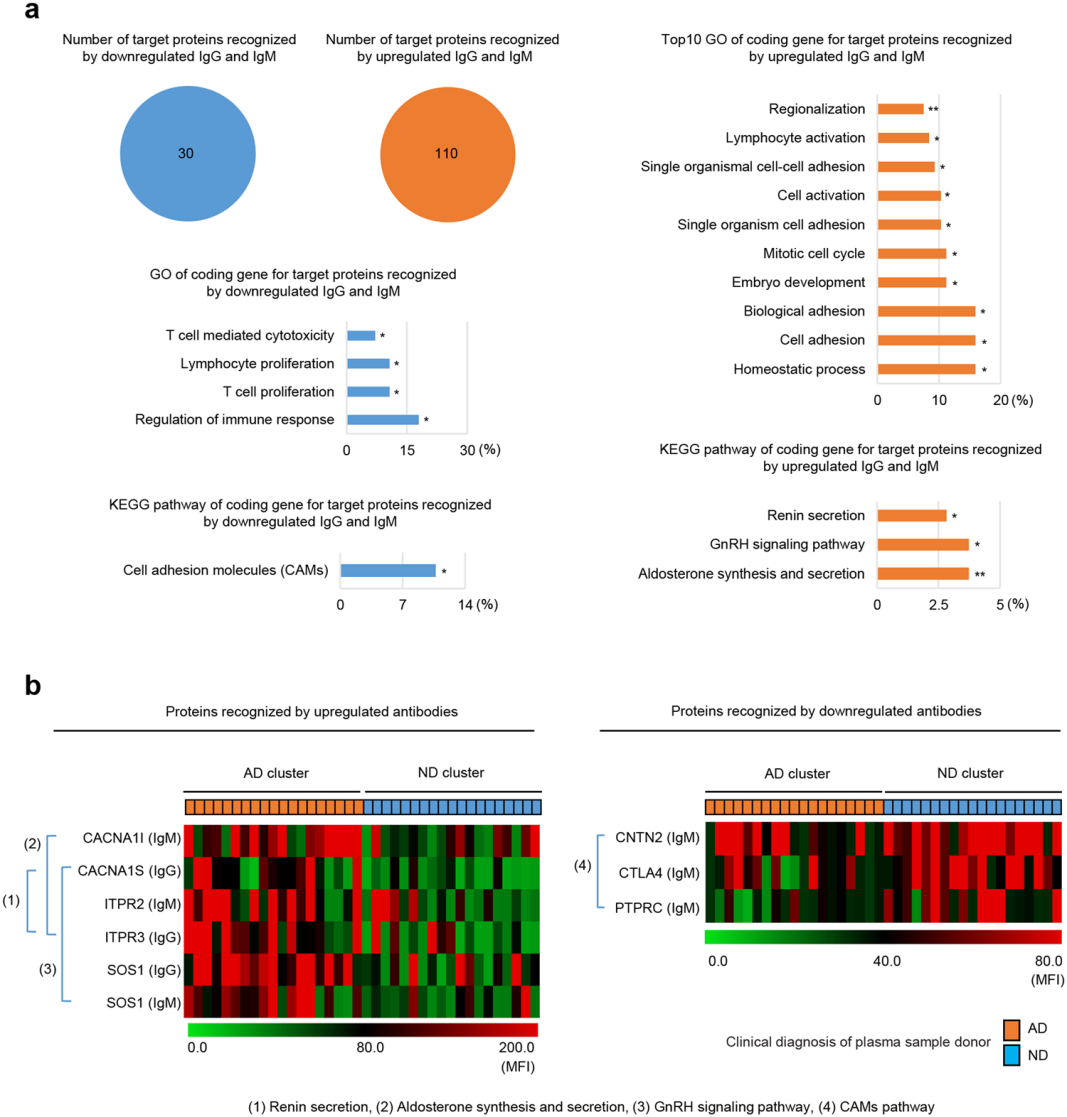
Gene functional classification of predicted target proteins recognized by antibodies differentially expressed in Alzheimer’s disease (AD). (**a**) Total number of target proteins recognized by downregulated IgG and IgM (blue circle), and by upregulated IgG and IgM (orange circle), antibodies in the plasma of AD patients. Gene ontology (GO) biological process and KEGG pathway categories involving downregulated IgG and IgM (left panel) and upregulated IgG and IgM (right panel) antibodies. All GO groups and KEGG groups were identified according to the EASE score (*P* ≤ 0.05). Bars represent the proportion of genes in each category (both statistical significance and number of genes are indicated). *P ≤ 0.05; **P ≤ 0.01 (modified Fisher’s exact test). (**b**) Heatmap showing target proteins involved in KEGG pathways. Each number represents the KEGG pathway to which the gene(s) encoding the target protein(s) belongs: (1) Renin secretion; (2) aldosterone synthesis and secretion; (3) the GnRH signalling pathway; and (4) the CAMs pathway. Abbreviations: AD, Alzheimer’s disease; ND, non-demented.

We identified 110 “most probable” upregulated, and 30 downregulated, protein targets. Functional classification of these targets was performed using the Database for Annotation, Visualization and Integrated Discovery (DAVID, release 6.8). We identified 34 biological processes categories and three KEGG pathways that involved target proteins recognized by antibodies upregulated in AD. Also, we identified four biological processes and one KEGG pathway for proteins recognized by antibodies downregulated in AD (Fig. 4a). Interestingly, the identified KEGG pathways (the renin secretion, gonadotropin-releasing hormone (GnRH) signalling, and aldosterone synthesis and secretion pathways) are known to be related to AD (Fig. 4a,b).

In addition to functional classifications, we also analysed the relationship between the identified proteins and AD. We found that some potential proteins are associated with AD. These include SOS1, TNFRSF21, ATM and S100A1 (recognized by upregulated IgG), SOS1 and SP4 (recognized by upregulated IgM) and GNPAT and CNTN2 (recognized by downregulated IgM) (Table 2; Supplementary Table S2).

## Discussion

Although the blood-borne antibodies have been studied as potential novel plasma markers of AD, most studies have focused on antibodies specific for AD-associated proteins such as Aβ, tau protein and glia markers^9^. A previous study that screened for AD-specific antibodies used human proteins^13^. In that study, around 9,000 proteins were used to profile IgG antibodies; the selected antibodies detected AD and AD-associated mild cognitive impairment patients. However, protein structures can be altered during storage and assay. Thus, peptides may have advantages over proteins with respect to standardization of diagnostic assays and increasing probe numbers. Besides, peptide screening may identify hidden epitopes associated with disease pathology that cannot be detected by protein screening methods^14,15^. Here, we used a peptide microarray with 29,240 random peptides to identify antibody-binding epitopes associated with AD. In addition to IgG isotype antibodies, we also examined IgM isotypes, thereby enabling identification of antibody class switching in AD.

We identified several characteristics of plasma antibodies associated with AD. First, IgG antibodies may be derived from established IgM-secreting cells through class switching, as AD-associated IgG-related fluorescence signals are already high in IgM-related fluorescence signals even in the ND group. Thus, the antibodies are already present in non-symptomatic people. However, pathologic conditions may trigger class switching. Actually, natural IgG is produced by B-2 B cells through class switching through a T cell-dependent process^10,11^. A study based on Genome-Wide Pathway Analysis shows that five genes (*HLA-DRB1*, *HLA-DRA*, *HLA-DOB*, *HLA-DQA1* and *TAP2*) involved in antigen processing and presentation pathways are associated with AD risk^26^. Therefore, we suggest the possibility that T cell-related immune interactions with antigenic proteins at the stage of class switching upregulate IgG in AD. In particular, we speculate that alterations in antigenic proteins at the AD stage render them recognizable by not only B cells but also T cells; thus IgM in healthy individuals undergoes class switching to generate IgG at the AD stage. This suggests that further study of alterations occurring in specific antigenic proteins, and their interaction with T cells during AD, is warranted.

Second, we identified proteins involved in several AD-related pathways. During *in silico* analysis, the peptide epitope profile was converted into a protein profile, which was then used for functional classification analysis. Thus, we identified 38 biological process-related pathways and four KEGG pathways. Interestingly, some studies report a relationship between these identified KEGG pathways and AD. For example, a previous study shows that luteinizing hormone, which is stimulated by GnRH, has a significant impact on Aβ40 and 42 levels^27^. A GnRH agonist, leuprolide acetate, has been tested in phase 2 clinical trials of late-onset AD; the results showed that high dose Lupron in combination with acetylcholinesterase inhibitors reduces cognitive decline in women^28^. Additionally, another recent report showed single-nucleotide polymorphisms of APOE and MS4A6A interact with GnRH signaling in AD^29^. In the case of the renin-angiotensin signalling (RAS) pathway, a large clinical study revealed that treatments of angiotensin receptor blockers and angiotensin-converting enzyme inhibitors show inverse dose-response relationships with AD^30^. Additionally, a clinical study used a RAS blocker to show that the blocker regulates glucose homeostasis and serum adipokines, thereby reducing the scores on the revised version of Hasegawa’s dementia scale^31^. The present study also suggests that these pathways may play roles in AD pathology, and we propose that these may be linked to production and class switching of antibodies that target these proteins. However, because the mechanism by which antibodies specific for these target proteins are regulated is unclear, it is necessary to investigate the mechanism underlying alterations in antibodies specific for these target proteins and their function during AD progression.

A relationship between several target proteins and AD has been reported. Interestingly, of these AD-related proteins, SOS1 expression is increased in the pyramidal neurons of AD patients^32^ and TNFRSF21 protein triggers neuronal death by binding to APP^33^. The ATM protein interacts with ATBF1, thereby mediating neuronal death via Aβ42^34^. Knocking out S100A1 reduces both plaque number and load^35^, and expression of SP4 protein (increased in the brain of AD patients) is associated with NFT and neuronal apoptosis^36^. By contrast, expression of GNPAT (a candidate protein recognized by downregulated IgM) is decreased in brains of AD model mouse and AD patients^37^. CNTN2 is also decreased in the brain of AD patients and correlates with expression of BACE1^38^. These results show that one factor underlying regulation of antibodies could be changes in expression of antigenic proteins. In particular, upregulation of antibodies in AD could be related to overexpression of antigenic proteins associated with aggravated AD pathology, whereas downregulation of antibodies in AD could be related to inhibition of antigenic protein expression (Supplementary Table S2). In addition, interestingly, the majority of candidate proteins still have not been reported as associated with AD. Therefore, these results may facilitate identification of novel mechanisms underlying AD pathogenesis.

In summary, the data presented herein suggest specific regulation of antibodies in AD, and that the peptides selected could be useful biomarkers for AD diagnosis and progression. These antibodies are both sequence- and isotype-specific and show certain patterns with respect to changes in isotype. This isotype-dependent regulation of antibodies is mediated by antigenic AD-related proteins and inflammatory immune responses. Thus, we have identified potential diagnostic probes (e.g., peptides), known AD-related known pathways and provided clues to the mechanisms underlying AD pathology.

## Methods

### Study populations

Plasma samples were collected from AD patients (n = 19) and age-matched ND individuals (n = 19) attending Chosun University Hospital and Chonnam National University Medical School in Korea. A clinical diagnosis of AD was based on the criteria of the National Institute on Aging-Alzheimer’s Association^39^. To confirm a diagnosis of AD, all patients and ND control subjects underwent magnetic resonance imaging or amyloid positron emission tomography imaging using F-18 florbetaben (NeuraCeq™). Korean Mini-Mental State Examination scores were measured to assess severity of dementia. The research was approved by the GIST institutional review board (20170823-HR-30-04-04). All subjects provided informed consent according to ethical guidelines for academic research and all experiments were performed in accordance with the relevant guidelines and regulations.

### Random peptide microarray

The random peptide microarray chips were printed with 29,240 random 15-mer peptides in duplicate (58,480 peptide spots). Microarrays also contained FLAG (DYKDDDDKAS) and HA (YPYDVPDYAG) as control peptides. Microarray chips were pre-swelled in PBS (pH 7.4)/0.05% Tween 20 and then blocked for 30 min with blocking buffer (Rockland, Gilbertsville, PA, USA; MB-070). The chips were then incubated for 45 min at room temperature with goat anti-human IgG (Fc) conjugated to DyLight680 and goat anti-human IgM (μ-chain) conjugated to DyLight800 (both at a dilution of 1:5,000) to analyse background binding between the secondary antibodies and the peptide library. The microarrays were then reacted for 16 h at 4 °C with plasma samples (diluted 1:500 in PBS (pH 7.4)/0.05% Tween 20/10% Rockland blocking buffer MB-070). Next, the chips were stained with goat anti-human IgG (Fc) conjugated to DyLight680 and goat anti-human IgM (μ-chain) conjugated to DyLight800 (1:5,000). Finally, internal FLAG and HA internal control peptides were stained for 45 min at room temperature with a monoclonal anti-HA antibody conjugated to DyLight680 and a monoclonal anti-FLAG antibody conjugated to DyLight800 to confirm the quality and integrity of the assay. After each incubation step, the microarray was washed three times with PBS (pH 7.4)/0.05% Tween 20. Data read-out was performed using a LI-COR Odyssey Imaging System at scanning intensities of 7/7 (red/green).

### Analysis of data generated by the random peptide microarray

Microarray data were processed using the following quality control procedure. First, the microarray data were evaluated using Spotxel® Microarray Image and Data Analysis software to remove background noise. Before normalizing the microarray data, the background signals generated by binding of secondary antibodies to peptides on the array were subtracted using a simple background subtraction method based on averaged median intensities. Normalization of raw data between arrays was performed using the quantile method and the *devtools* package in R 3.4.0 software (R Development Core Team, 2017). Peptides bound to antibodies differentially expressed in AD were pre-selected according to -fold changes (≥2) and *P*-values (calculated using Student’s t-test; *P* ≤ 0.05). Pre-selected peptides were ranked according to the area under the receiver operating characteristic curve (AUC), which provides a summary statistic of diagnostic performance, using the *pROC* package in R 3.4.0 software (R Development Core Team, 2017). The top 143 peptides with a threshold AUC value ≥0.75 were selected. Hierarchical clustering analysis, combined with heatmaps of the top selected peptides, was performed using Multiple Experiment Viewer (version 4.9.0).

### Protein prediction analyses

Epitopes recognized by antibodies were predicted using BepiPred-2.0 (http://www.cbs.dtu.dk/services/BepiPred/), with a user-defined threshold of 0.4 (based on a random forest algorithm trained using epitopes derived from antibody-antigen protein structures)^24^. The identified epitopes were used to predict potential antigenic proteins using the Basic Local Alignment Search Tool (BLAST) from the National Center for Biotechnology Information, which is a protein sequence similarity search program, and the UniProtKB/Swiss-Prot (Swiss-Prot) database (organism humans (taxid: 9605)). BLAST parameters were set as follows: PAM30 matrix, gap open 9, gap extend 1, threshold 11, window size 40, word size 2. Candidate proteins corresponding to each identified epitope were ranked according to high identity values, assigning a value of +1 for consecutive matched amino acids and +0.5 for conserved amino acids. The top protein was then selected.

### Gene functional classification

Gene functional classification was performed using the Database for Annotation, Visualization and Integrated Discovery (DAVID, release 6.8) (https://david.ncifcrf.gov). EASE scores (*P* ≤ 0.05) were used to identify gene enrichment of functional pathways by estimating the number of genes belonging to functional pathways involving the selected target proteins.

### Enzyme-linked immunosorbent assay (ELISA)

Total plasma IgG (Innovative Research, Novi, MI, USA; IHUIGGKT) and IgM (Innovative Research, IHUIGMKT) were measured using commercial enzyme-linked immunosorbent assay (ELISA) kits, according to the manufacturer’s protocols.

### Cytokine measurement

The concentrations of IL-12p70, TNF, IL-10, IL-6, IL-1β and IL-8 in plasma were measured using a BD Cytometric Bead Array human inflammatory cytokines kit (BD Biosciences, San Jose, CA, USA; #551811), according to the manufacturer’s protocols. A FACSCanto II flow cytometer (BD Biosciences) was used to acquire samples, and data were analysed using FCAP array software v3.0 (BD Biosciences).

### Statistical analysis

The significance of differences between two groups was analysed using Student’s t-test. Receiver operating characteristic curve analysis of the top peptides and calculation of Pearson’s correlation coefficient were performed using OriginPro9.1 software (Origin Lab Corporation). All data are expressed as the mean ± SD. For all statistical tests, *P*-values ≤ 0.05 were considered significant.

## Supplementary information

Supplementary information

## Data Availability

Datasets generated and/or analysed during the current study are available from the corresponding author upon reasonable request.
