# Supplementary material for: High-throughput epitope profiling of antibodies in the plasma of Alzheimer’s disease patients using random peptide microarrays
Source: Sci Rep. 2019 Mar 14;9:4587. doi: 10.1038/s41598-019-40976-x (PMC6418098; doi:10.1038/s41598-019-40976-x)
Supplement: Supplementary file 1 — Supplementary information [file 41598_2019_40976_MOESM1_ESM.pdf]

Supplementary information for:

High-throughput epitope profiling of antibodies in the plasma of Alzheimer's disease patients using random peptide microarrays

Kyu-Young Sim, Sang-Heon Park, Kyu Yeong Choi, Jung Eun Park, Jung Sup Lee, Byeong C. Kim, Jeonghwan Gwak, Woo Keun Song, Kun Ho Lee, Sung-Gyoo Park

The file contains the following:

Supplementary Figure 1 and 2
Supplementary Table 1 and 2
Supplementary Reference

Supplementary Figures

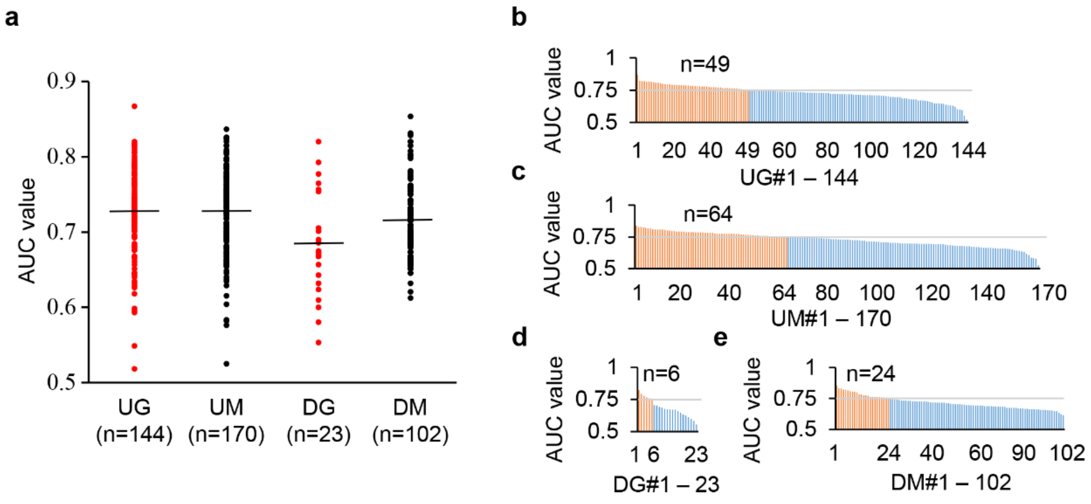

Supplementary Figure S1. Area under the receiver operating characteristic curve (AUC) data-based selection of peptides recognized by antibodies differentially regulated in Alzheimer's disease (AD). (a) Dot plots generated by AUC of peptides targeted by upregulated IgG and IgM, and by downregulated IgG and IgM, antibodies in AD plasma. (b-e) Selection of peptides recognized by upregulated IgG (b) and IgM (c) and downregulated IgG (d) and IgM (e) in AD plasma. Red denotes selected peptides (AUC ≥ 0.75) and blue denotes non-selected peptides (AUC < 0.75). For peptide ID: UG and UM indicate peptides recognized by upregulated IgG and IgM, respectively, and DG and DM indicate peptides recognized by downregulated IgG and IgM, respectively.

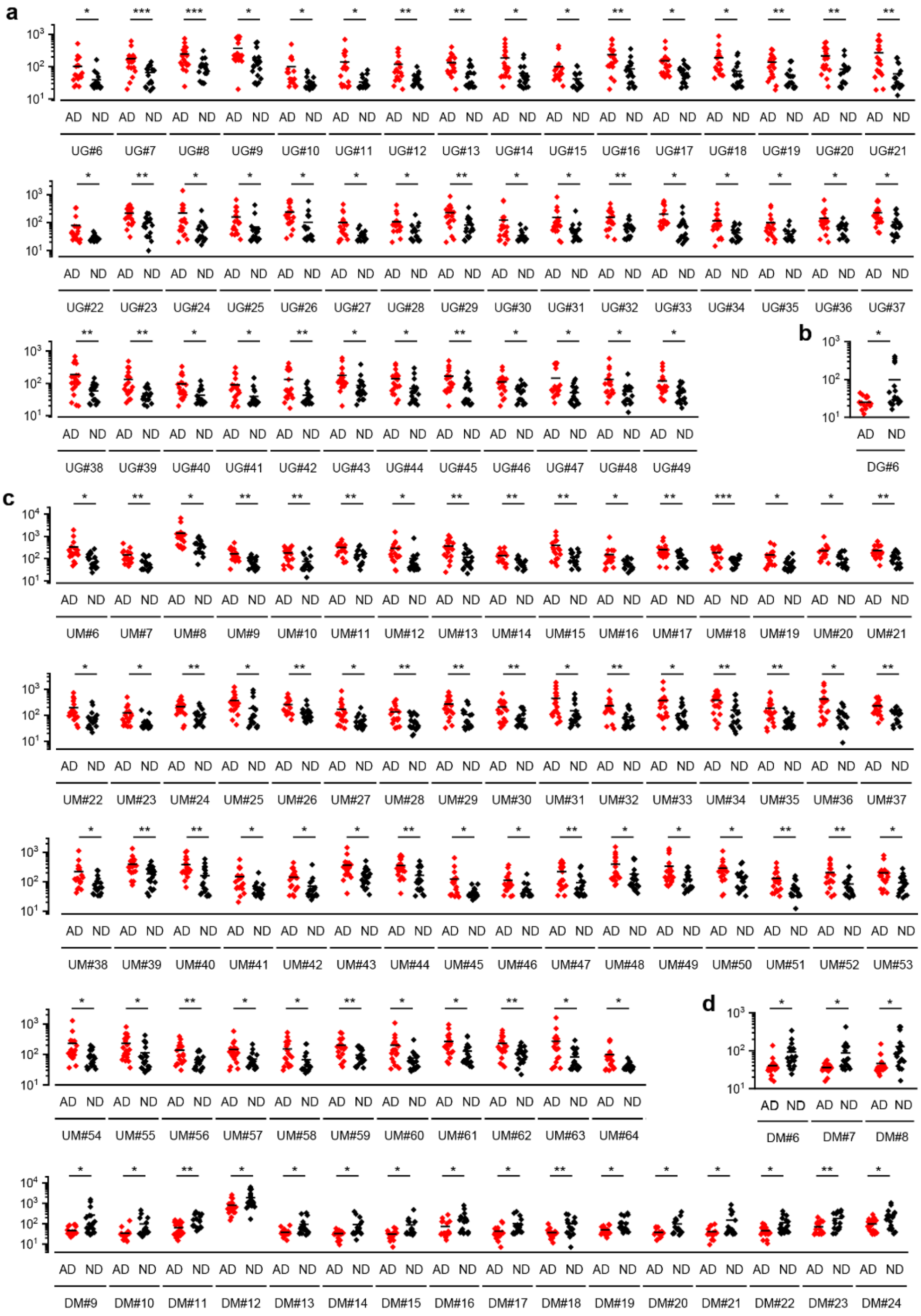

Supplementary Figure S2. Dot plots showing all selected peptides targeted by antibodies differentially expressed in AD plasma. (a–d) Each dot represents the median fluorescence intensity (MFI) of antibodies targeting selected peptides in the microarray in AD and ND. Dot plots show the MFI of upregulated IgG (a) and downregulated IgG (b), and upregulated IgM (c) and downregulated IgM (d), antibodies targeting each group of selected peptides (ranked according to AUC value ≥ 0.75). *, $P \leq 0.05$; **, $P \leq 0.01$; and ***, $P \leq 0.001$ (Student's t-test). For peptide ID: UG and UM indicate peptides recognized by upregulated IgG and IgM, respectively, and DG and DM indicate peptides recognized by downregulated IgG and IgM, respectively.

Supplementary Tables

Supplementary Table 1. Diagnostic utility of the peptides targeted by antibodies differentially expressed in AD

Peptide ID	Peptide sequence	AUC	CoE of peptide with K-MMSE score (*P*-value)	CoE of peptide with total plasma IgG or IgM (*P*-value)
UG#1	PPHVISYHSYPDAWD	0.867*	-0.218† (0.189‡)	0.021† (0.902‡)
UG#2	SKGTDPTRTVWERPF	0.820*	-0.248† (0.134‡)	0.116† (0.487‡)
UG#3	FLDMDQRPWNPFVWD	0.817*	-0.239† (0.149‡)	-0.083† (0.619‡)
UG#4	EHPHRDELQQRFFPD	0.817*	-0.273† (0.097‡)	0.084† (0.615‡)
UG#5	SNLIRLKEFTDYLFF	0.814*	-0.31† (0.058‡)	0.317† (0.053‡)
UG#6	RPLDYRVWEVNWDHT	0.814*	-0.296† (0.071‡)	0.298† (0.069‡)
UG#7	RIFWPTAREFDFSEW	0.812*	-0.579† (<0.001‡)	0.122† (0.467‡)
UG#8	IHKKFPEFGEDWWIF	0.809*	-0.315† (0.054‡)	0.109† (0.516‡)
UG#9	NHSACFDQFAWFFDF	0.806*	-0.415† (0.01‡)	0.1† (0.551‡)
UG#10	MPSDREQSLAEASFS	0.805*	-0.108† (0.52‡)	0.215† (0.196‡)
UG#11	NQLVFDCHVAFMFVV	0.801*	-0.237† (0.152‡)	0.007† (0.965‡)
UG#12	RIEKRFKPDYMDCWG	0.798*	-0.2† (0.23‡)	-0.075† (0.657‡)
UG#13	GDPVDTVGMDAVIHY	0.795*	-0.316† (0.053‡)	0.036† (0.829‡)
UG#14	HPYPMHQFIENNMYV	0.792*	-0.271† (0.1‡)	0.231† (0.162‡)
UG#15	HEQHNTRSFEGYDNC	0.792*	-0.488† (0.002‡)	0.18† (0.278‡)
UG#16	WEWDRQDFFPSMQWD	0.789*	-0.437† (0.006‡)	-0.097† (0.564‡)
UG#17	KWRYNQESYVQWFEF	0.789*	-0.26† (0.115‡)	-0.055† (0.745‡)
UG#18	IHGDFTLDMYERFFV	0.789*	-0.243† (0.142‡)	0.245† (0.137‡)
UG#19	NYSWPYIDPIVAAWD	0.787*	-0.364† (0.025‡)	0.06† (0.721‡)
UG#20	MFNPWCAQDAYADEF	0.787*	-0.581† (<0.001‡)	0.144† (0.387‡)
UG#21	FIWFMIDDDSVSSWM	0.787*	-0.359† (0.027‡)	0.027† (0.873‡)
UG#22	NMGDTFLGVKIQMMD	0.784*	-0.214† (0.196‡)	-0.078† (0.644‡)
UG#23	FAMQPIFDWYVPFEW	0.784*	-0.534† (0.001‡)	0.25† (0.131‡)
UG#24	EFFDHMFDHPLFDKD	0.781*	-0.22† (0.185‡)	-0.175† (0.293‡)
UG#25	NVGDNIPDLQNSDWS	0.778*	-0.223† (0.179‡)	0.097† (0.56‡)
UG#26	MTTRGDADWDDERWH	0.778*	-0.514† (0.001‡)	0.101† (0.544‡)
UG#27	KQYLTRQESIPYWMD	0.778*	-0.263† (0.11‡)	-0.188† (0.257‡)
UG#28	WNTGDHIEYWEFPDT	0.776*	-0.075† (0.653‡)	-0.15† (0.369‡)
UG#29	NESWSGWNGVWHFFD	0.776*	-0.388† (0.016‡)	0.024† (0.886‡)
UG#30	LDWRRVNVYRFFYTD	0.776*	-0.144† (0.388‡)	0.157† (0.346‡)
UG#31	HNDKFMDKPDTPDFC	0.776*	-0.263† (0.11‡)	0.009† (0.956‡)
UG#32	TNHVQWDQCTIWGDF	0.771*	-0.614† (<0.001‡)	0.17† (0.308‡)
UG#33	WPDEMVPIWFHYMHY	0.770*	-0.452† (0.004‡)	0.184† (0.269‡)
UG#34	GERIHLKHFDIAVFD	0.770*	-0.456† (0.004‡)	0.296† (0.071‡)
UG#35	EFYICDTMTQWWHEQ	0.770*	-0.128† (0.443‡)	-0.234† (0.158‡)
UG#36	NMDASEYHRWPMREF	0.767*	-0.336† (0.039‡)	-0.082† (0.623‡)
UG#37	FENDNFFWEVWVDRW	0.767*	-0.358† (0.027‡)	0.039† (0.817‡)
UG#38	SHDFEGAADTQCVWD	0.765*	-0.359† (0.027‡)	0.026† (0.876‡)
UG#39	NVYHDNSVSDEWCWA	0.765*	-0.185† (0.266‡)	-0.135† (0.42‡)
UG#40	NHPEYDTLRPLGQWS	0.765*	-0.215† (0.194‡)	-0.086† (0.609‡)
UG#41	FSQGHMFGEWYANDM	0.763*	-0.194† (0.244‡)	0.316† (0.053‡)
UG#42	NSPMLFPMVFCWMLI	0.762*	-0.431† (0.007‡)	0.185† (0.266‡)
UG#43	NQNEADALRGFEMDW	0.762*	-0.435† (0.006‡)	0.167† (0.315‡)
UG#44	HISMEYFGNVDHPVW	0.762*	-0.302† (0.065‡)	0.045† (0.788‡)
UG#45	WQAFQMQENWDFQFD	0.759*	-0.524† (0.001‡)	0.071† (0.671‡)
UG#46	RTNFHVPPDWWLAWD	0.756*	-0.655† (<0.001‡)	0.188† (0.258‡)

UG#47	NMINMGKTADHWMLW	0.753*	-0.261† (0.114‡)	-0.052† (0.755‡)
UG#48	NVANEPSSFFISFCY	0.751*	-0.272† (0.099‡)	-0.056† (0.738‡)
UG#49	NFMYMDVYYWTVAAF	0.751*	-0.382† (0.018‡)	-0.152† (0.362‡)
UM#1	RGPLRQGRNTGAAGA	0.837*	-0.509† (0.001‡)	0.054† (0.745‡)
UM#2	VFTAWMGLEFAYGDK	0.825*	-0.325† (0.047‡)	-0.221† (0.183‡)
UM#3	HKRIFGSHHPTHDLG	0.825*	-0.185† (0.267‡)	-0.172† (0.303‡)
UM#4	GRVWNVDMRRCDFSE	0.823*	-0.371† (0.022‡)	0.283† (0.085‡)
UM#5	GQRDAPMVTHRPSPH	0.820*	-0.358† (0.027‡)	-0.231† (0.163‡)
UM#6	PYHDCDQNFADWPGV	0.814*	-0.191† (0.25‡)	-0.023† (0.892‡)
UM#7	APYNTFHFGRILPEG	0.814*	-0.25† (0.131‡)	-0.283† (0.085‡)
UM#8	HVIHGPRYGECEEFF	0.809*	-0.163† (0.327‡)	0.253† (0.125‡)
UM#9	VVVVEHFDKFAWGER	0.806*	-0.251† (0.129‡)	-0.133† (0.427‡)
UM#10	RMDKNPLAKIHHSEF	0.806*	-0.428† (0.007‡)	0.004† (0.982‡)
UM#11	PDRNSGRACADTDEF	0.806*	-0.252† (0.127‡)	-0.191† (0.251‡)
UM#12	GQPIWSTDYLMDSTN	0.806*	-0.027† (0.874‡)	-0.145† (0.385‡)
UM#13	YEMTTDRHMPEFTVC	0.803*	-0.389† (0.016‡)	-0.193† (0.246‡)
UM#14	RYHDRSIHPDRWVCN	0.798*	-0.277† (0.092‡)	0.113† (0.498‡)
UM#15	RWDITIGDMSSADCP	0.798*	-0.301† (0.066‡)	-0.062† (0.71‡)
UM#16	YKVTGTEKDDTPDFT	0.795*	-0.067† (0.69‡)	-0.11† (0.512‡)
UM#17	ARAPHTYYVWFFPFE	0.795*	-0.169† (0.311‡)	0.117† (0.485‡)
UM#18	IKVSYMWLFWIDPMH	0.789*	-0.247† (0.135‡)	-0.075† (0.656‡)
UM#19	HNHTRRNVYKWFCEG	0.789*	-0.315† (0.054‡)	0.186† (0.264‡)
UM#20	HDEPARHPSQVLWGD	0.789*	-0.386† (0.017‡)	-0.116† (0.488‡)
UM#21	GPCDTFSWWEPKDFQ	0.789*	-0.354† (0.029‡)	0.052† (0.757‡)
UM#22	PDTMFHTMGVGHHYQ	0.787*	-0.048† (0.773‡)	-0.112† (0.502‡)
UM#23	HHNVVLPMMANGFTD	0.787*	-0.217† (0.191‡)	-0.073† (0.664‡)
UM#24	DQCKDLEPRPPRFRE	0.787*	-0.415† (0.01‡)	0.014† (0.931‡)
UM#25	AIMDKFYQGDPDMTN	0.787*	-0.067† (0.69‡)	-0.075† (0.653‡)
UM#26	VERPETHAGYMMDNF	0.784*	-0.604† (<0.001‡)	-0.295† (0.072‡)
UM#27	QWLTSHCDGPVLESM	0.784*	-0.201† (0.226‡)	0.208† (0.211‡)
UM#28	GSSIGCLHEAAPSNS	0.784*	-0.355† (0.029‡)	-0.203† (0.221‡)
UM#29	ASHAHLFDPTESAFF	0.784*	-0.347† (0.033‡)	-0.264† (0.109‡)
UM#30	GKAQSKIMNEDPLWV	0.780*	-0.35† (0.031‡)	-0.238† (0.15‡)
UM#31	SLFDVEMFYAHMEVI	0.778*	-0.087† (0.602‡)	-0.402† (0.012‡)
UM#32	MASHMSVPMHGDDAM	0.778*	-0.36† (0.026‡)	-0.043† (0.796‡)
UM#33	ICMYVKVRFFQPEGG	0.778*	-0.318† (0.052‡)	-0.146† (0.383‡)
UM#34	TYPQEIMGLPFMELV	0.776*	-0.238† (0.151‡)	-0.114† (0.497‡)
UM#35	NDVACRMNGGVPNDN	0.776*	-0.473† (0.003‡)	-0.096† (0.566‡)
UM#36	VYSLTMDLPGDLHIV	0.773*	-0.272† (0.098‡)	-0.039† (0.818‡)
UM#37	KLSVLRHGHPIDGYM	0.773*	-0.296† (0.071‡)	-0.133† (0.426‡)
UM#38	IPYPWILWSMYGDNT	0.773*	-0.311† (0.057‡)	-0.297† (0.07‡)
UM#39	HDAVEEPRYPQFPGY	0.773*	-0.043† (0.799‡)	-0.189† (0.256‡)
UM#40	FPNKDDKNVHNADFF	0.773*	-0.333† (0.041‡)	-0.148† (0.374‡)
UM#41	AEYSENSFEQHLDMS	0.773*	-0.18† (0.28‡)	-0.12† (0.474‡)
UM#42	AERDWANGHDCPQNR	0.773*	-0.339† (0.037‡)	-0.172† (0.301‡)
UM#43	KPQCEPDNPDDFLMF	0.770*	-0.125† (0.454‡)	0.124† (0.46‡)
UM#44	HNDKFMDKPDTPDFC	0.770*	-0.35† (0.031‡)	-0.185† (0.267‡)
UM#45	IYVHPWLWFKHTVEV	0.767*	-0.216† (0.192‡)	0.387† (0.016‡)
UM#46	IVWNCVPWIMGGPFS	0.767*	-0.323† (0.048‡)	0.236† (0.154‡)
UM#47	TTADEWNKVNEFFTF	0.765*	-0.178† (0.284‡)	-0.012† (0.944‡)
UM#48	CGFRRALSHFPDIFY	0.763*	-0.241† (0.146‡)	0.101† (0.546‡)
UM#49	GYTDREGPDNPIGIC	0.762*	-0.126† (0.45‡)	-0.098† (0.56‡)

UM#50	FATPYMWQPGIGDHM	0.762*	-0.436† (0.006‡)	-0.115† (0.491‡)
UM#51	HRGAAFTHDIEKWMV	0.759*	-0.219† (0.186‡)	-0.204† (0.22‡)
UM#52	CHQQGRVPGPGATRQ	0.759*	-0.227† (0.171‡)	0.142† (0.395‡)
UM#53	PDRWWELTIIENFVH	0.756*	-0.151† (0.366‡)	-0.306† (0.062‡)
UM#54	HHDDVAQRRIPGDRQ	0.756*	-0.438† (0.006‡)	-0.126† (0.449‡)
UM#55	GYTESMAPNKDKDYQ	0.756*	-0.255† (0.122‡)	-0.365† (0.024‡)
UM#56	DYEAMNQGMFQIFLD	0.756*	-0.215† (0.194‡)	-0.005† (0.978‡)
UM#57	SNLIRLKEFTDYLFF	0.753*	-0.077† (0.644‡)	-0.194† (0.243‡)
UM#58	QTLDNDPSTYMLVTF	0.753*	-0.174† (0.297‡)	-0.037† (0.826‡)
UM#59	PQTCKRFDCPTPIFF	0.753*	-0.23† (0.165‡)	-0.092† (0.583‡)
UM#60	AFGLNGHPQHSLCQC	0.753*	-0.122† (0.464‡)	-0.139† (0.406‡)
UM#61	RCGSLRYYNHDIPGD	0.751*	-0.289† (0.078‡)	0† (0.999‡)
UM#62	QHVTDNIHFLMYMDM	0.751*	-0.012† (0.943‡)	-0.142† (0.395‡)
UM#63	PGFCWHMMYPGLGEA	0.751*	-0.604† (<0.001‡)	0.356† (0.028‡)
UM#64	FAWMTDEPSGKKPMQ	0.751*	-0.221† (0.183‡)	-0.126† (0.45‡)
DG#1	DPVTRSEERGNSANA	0.820*	0.239† (0.148‡)	0.105† (0.531‡)
DG#2	GTFFEYDQIIRANKQ	0.792*	0.295† (0.072‡)	-0.376† (0.02‡)
DG#3	ENVAEETRMSSRDAG	0.777*	0.262† (0.112‡)	-0.217† (0.191‡)
DG#4	GKLWGPWTFTLTEDV	0.765*	0.225† (0.174‡)	0.171† (0.304‡)
DG#5	ESSMVWNAWDKMKAP	0.756*	0.323† (0.048‡)	-0.136† (0.416‡)
DG#6	RKDWTNHFEEGVATS	0.753*	0.366† (0.024‡)	-0.233† (0.158‡)
DM#1	ALNSNWSWRYFVRYS	0.853*	0.34† (0.037‡)	0.107† (0.524‡)
DM#2	DMFFETDGKKKRNKN	0.831*	0.298† (0.069‡)	-0.005† (0.974‡)
DM#3	QFALYQLQWWRFQLI	0.828*	0.261† (0.114‡)	0.141† (0.4‡)
DM#4	MYTKKEQWQEQKFQP	0.820*	0.252† (0.127‡)	0.153† (0.36‡)
DM#5	AWYNPMFQNNNFFQP	0.820*	0.378† (0.019‡)	0.016† (0.926‡)
DM#6	KIWLNRWYSKEITLY	0.814*	0.29† (0.077‡)	0.143† (0.393‡)
DM#7	EWRFVHYKQYNIKIM	0.807*	0.227† (0.171‡)	0.173† (0.299‡)
DM#8	VKLWVNWTHHQTSQY	0.803*	0.378† (0.019‡)	0.017† (0.922‡)
DM#9	KEQRAFFLIVYHAFP	0.801*	0.223† (0.178‡)	-0.022† (0.897‡)
DM#10	TSRWFTWIVTEPAAK	0.798*	0.312† (0.056‡)	-0.175† (0.293‡)
DM#11	GINFCSWFEKNTKAV	0.781*	0.275† (0.095‡)	-0.03† (0.857‡)
DM#12	TAIHNMDECEEEEEK	0.778*	0.263† (0.111‡)	0.189† (0.255‡)
DM#13	AQEVMYPINHTTYWS	0.776*	0.315† (0.054‡)	-0.09† (0.591‡)
DM#14	KHQAYQRKNRYVPVN	0.773*	0.342† (0.035‡)	-0.231† (0.162‡)
DM#15	YRERSAEIMCKSVPI	0.770*	0.265† (0.108‡)	-0.011† (0.949‡)
DM#16	PVLPMRSWDAAQVMK	0.762*	0.267† (0.105‡)	-0.117† (0.485‡)
DM#17	IQSIIQIMCQTFKYS	0.762*	0.286† (0.081‡)	-0.122† (0.466‡)
DM#18	HNKKLITNFNIFQQP	0.759*	0.384† (0.017‡)	-0.178† (0.286‡)
DM#19	FRQYATDEYLETQQA	0.759*	0.347† (0.033‡)	-0.07† (0.676‡)
DM#20	EGPMYKKKKMFSGMT	0.759*	0.265† (0.107‡)	0.153† (0.358‡)
DM#21	FWGIYSCFRWKIFPS	0.756*	0.163† (0.329‡)	0.099† (0.552‡)
DM#22	WKAALNMATRTRYAA	0.755*	0.258† (0.119‡)	0.057† (0.735‡)
DM#23	EVYQFQFPWMNYPLT	0.753*	0.278† (0.091‡)	-0.01† (0.953‡)
DM#24	YSPVKLEYMWYQENM	0.751*	0.28† (0.089‡)	-0.011† (0.948‡)

For peptide ID, UG and UM indicate peptides recognized by upregulated IgG and IgM, respectively, DM indicate peptides recognized by downregulated IgM; data are shown as the AUC* value or as the Pearson correlation coefficient† (*P*-value‡). Pearson correlation coefficient was analyzed to determine the statistical significance of the correlation.

Abbreviations: AUC, area under the receiver operating characteristic curve; CoE, correlation coefficient; K-MMSE, Korean Mini-Mental State Examination.

Supplementary Table 2. Selection of potential antigenic proteins by epitope prediction analysis and BLAST,

Peptide ID	Predicted epitope	Subject	Identity	Protein name	Sequence ID	Association with AD
UG#1	ISYHSYPD	IS+HSY	5.5	PORCN	Q9H237.2	N/A
UG#2	TDPTRTVWER	RTVWER	6	GBA2	Q9HCG7.2	N/A
UG#3	DMDQRPWNPF	RPWNP	5	PD-1	Q15116.3	Blockade of PD1 inhibits AD pathology in a mouse model [3]
UG#4	HRDELQQRFF	HRDELQ	6	FAM83D	Q9H4H8.3	N/A
UG#5	IRLKEFTDYL	KEFTDYL	7	SOS1	Q07889.1	Increased expression in pyramidal neurons of AD patients [4]
UG#6	DYRVWEVNW	RVW+VN	5.5	BTRC	Q9Y2P7.1	N/A
UG#7	WPTAREFDFS	TAREFD	6	BIG3	Q5TH69.3	N/A
UG#8	KKFPEFGEDWW	KFPE+GE	6.5	TMC3	Q7Z5M5.3	N/A
UG#9	CFDQFAWF	CFDQF	5	CYFIP2	Q96F07.2	Reduced in AD patients and in a mouse model; associated with Aβ42 production [5]
UG#10	SDREQSLAEA	SDR+QSL	6.5	SorCS2	Q96PQ0.3	SNP is associated with AD [6]
UG#11	FDCHVAFM	FDCHV	5	AHDC1	Q5TGY3.1	N/A
UG#12	EKRFKPDYMDC	RFKP+Y	5.5	ZIP4	O14863.2	N/A
UG#13	VDTVGMDAV	DTVGMD	6	ALX4	Q9H161.2	N/A
UG#14	HQFIENNM	HQFIEN	6	RAB4B	P61018.1	N/A
UG#15	QHNTRSFEGYD	HNTRSF	6	MUC3A	Q02505.3	N/A
UG#16	WDRQDFFPSMQ	DR+DFF	5.5	CNBD2	Q96M20.2	N/A
UG#17	RYNQESYVQWF	YNQ+SY	5.5	SYNPR	Q8TBG9.1	N/A
UG#18	DFTLDMYER	+FTLDM	5.5	ITPR3	Q14573.2	N/A
UG#19	PYIDPIVA	IDP+VA	5.5	TRIP12	Q14669.1	N/A
UG#20	WCAQDAYAD	CAQDA+	5.5	ECH1	Q13011.2	Included in new AD blood biomarker panels [7]
UG#21	MIDDDSVSS	DDSVSS	6	RANBP2	P49792.2	N/A
UG#22	FLGVKI	FLGVK	5	SIN3B	Q75182.2	N/A
UG#23	QPIFDWYVP	QPIFD	5	TNFRSF21	Q75509.1	Triggers neuronal death by binding to APP [8]
UG#24	DHMFDHPLF	MF+HPL	5.5	PRKDC	P78527.3	N/A
UG#25	DNIPDLQNSD	DNIP+L	5.5	GPR98	Q8WXG9.2	N/A
UG#26	RGDADWDDER	DA+WDD	5.5	LRP4	O75096.4	N/A
UG#27	YLTRQESIPYW	LTRQES	6	PARG	Q86W56.1	N/A
UG#28	GDHIEYWEFP	GDHIEY	6	OXA1L	Q15070.3	N/A
UG#29	WSGWNGVWH	NGVWH	5	VWA8	A3KMH1.2	N/A
UG#30	RRVNVYRFF	RVNV+R	5.5	NAT10	Q9H0A0.2	N/A
UG#31	KFMDKPDTPD	FMDKP+	5.5	ICE2	Q659A1.2	N/A
UG#32	VQWDQCTIW	QWD+CT	5.5	ATM	Q13315.4	Interacts with ATBF1; mediates neuronal cell death via Aβ42 [9]
UG#33	MVPIWFHY	PIWFH	5	G6PC	P35575.2	N/A
UG#34	RIHLKHFDIA	LKHFDI	6	CACNA1S	Q13698.4	N/A
UG#35	ICDTMTQWWH	CDTMT	5	POLR2A	P24928.2	N/A
UG#36	ASEYHRWPMR	SEYHR	5	POLE	Q07864.5	N/A
UG#37	DNFFWEVWV	+NFFWE	5.5	S100A1	P23297.2	Knockout decreases plaque number and load [10]
UG#38	FEGAADTQCV	GAADTQ	6	CD3D	P04234.1	N/A
UG#39	YHDNSVSDEWC	DNSVSD+	6.5	REV3L	O60673.2	N/A
UG#40	EYDTLRPLGQ	EY+TLR	5.5	DST	Q03001.4	N/A
UG#41	GHMFGEWYA	HMFG+	4.5	WDTC1	Q8N5D0.2	N/A
UG#42	N/A					N/A
UG#43	EADALRGFEM	EA+ALRG	6.5	MYO5A	Q9Y4I1.2	Altered in the CA region of

						AD patients [11]
UG#44	MEYFGNVDHP	+YFGNV	5.5	PHEX	P78562.1	N/A
UG#45	FQMQENWDFQ	+QENWD	5.5	PAPPA	Q13219.3	N/A
UG#46	FHVPPDWWL	HVPP+W	5.5	VCPIP1	Q96JH7.2	N/A
UG#47	MGKTADHWM	MGKTA+	5.5	DDX10	Q13206.2	N/A
UG#48	NEPSSFFIS	NEPSSF	6	C9orf84	Q5VXU9.1	N/A
UG#49	MDVYYWT	MDVYY	5	LRP1B	Q9NZR2.2	Gene is associated with AD in Caribbean Hispanic individuals [12]
UM#1	PLRQGRNTGAA	PLRQGR	6	BUB1b	O60566.3	N/A
UM#2	AWMGLEFAY	GLEFAY	6	SLC15A3	Q8IY34.2	N/A
UM#3	IFGSHHPTHD	SHHPT	5	PTPRD	P23468.2	Locus is associated with NFT [13]
UM#4	WNVDMRRCDF	VD+RRC	5.5	ARHGAP9	Q9BRR9.2	N/A
UM#5	DAPMVTHRPS	+VTHRPS	6.5	COL6A5	A8TX70.1	N/A
UM#6	DCDQNFADWP	+QNFAD	5.5	CUL9	Q8IWT3.2	N/A
UM#7	NTFHFGRIL	NTFHF	5	POLR1A	O95602.2	N/A
UM#8	HGPRYGECEE	GPRYGE	6	CDK13	Q14004.2	N/A
UM#9	VEHFDKFAWG	+EHFDK	5.5	SLC39A10	Q9ULF5.2	N/A
UM#10	KNPLAKIHHS	K+PLAK	5.5	OLFML2B	Q68BL8.2	N/A
UM#11	RNSGRACADTD	SGRACA+	6.5	CDO	Q9BWV1.1	N/A
UM#12	IWSTDYLMD	WSTDY	5	ADAM29	Q9UKF5.3	N/A
UM#13	MTTDRHMPEFT	RHMPE	5	GUCY2F	P51841.2	N/A
UM#14	HDRSIHPDRWV	RSIHPD	6	WDR81	Q562E7.2	N/A
UM#15	ITIGDMSSAD	IGDMSS	6	GLI3	P10071.6	Variants are associated with language performance in AD [14]
UM#16	TGTEKDDTPD	EKDDTP	6	CBLL1	Q75N03.1	N/A
UM#17	YYVWFF	YYVWF	5	SLC35A1	P78382.1	N/A
UM#18	YMWLFWI	Y+WLFW	5.5	DISP1	Q96F81.3	N/A
UM#19	TRRNVYKWFC	RR+VYK	5.5	CFAP47	Q6ZTR5.4	N/A
UM#20	EPARHPSQVLW	HPSQVL	6	CDON	Q4KMG0.2	N/A
UM#21	DTFSWWEPKD	WEPKD	5	CHD5	Q8TDI0.1	N/A
UM#22	FHTMGVGH	FHTMG	5	NCOA2	Q15596.2	N/A
UM#23	N/A					N/A
UM#24	CKDLEPRPPRF	EPRPPR	6	DACT3	Q96B18.2	N/A
UM#25	DKFYQGDPDM	KF+QGD	5.5	OR52N5	Q8NH56.2	N/A
UM#26	PETHAGYMM	P+THAG	5.5	KIAA1958	Q8N8K9.1	N/A
UM#27	TSHCDGPVL	CDGPVL	6	ACTRT3	Q9BYD9.1	N/A
UM#28	IGCLHEAAPS	LHEAAPS	7	C1orf198	Q9H425.1	N/A
UM#29	AHLFDPTESA	HLFDPT	6	CFAP61	Q8NHU2.3	N/A
UM#30	QSKIMNEDPL	QSK+MN	5.5	GKAP1	Q5VSY0.2	N/A
UM#31	VEMFYAH	EMFYA+	5.5	KCTD19	Q17RG1.1	N/A
UM#32	MSVPMHGD	PMHGD	5	MARCH4	Q9P2E8.2	N/A
UM#33	YVKVRFFQPE	RFF+PE	5.5	FUCA1	P04066.4	N/A
UM#34	QEIMGLPFM	QE+MGLP	6.5	ZBTB5	O15062.1	N/A
UM#35	CRMNGGVPN	CR+NGG	5.5	DUSP15	Q9H1R2.4	N/A
UM#36	LTMDLPGDLH	DLPG+LH	6.5	PATJ	Q8NI35.3	N/A
UM#37	VLRHGHPIDG	HGHPID	6	TRIM59	Q8IWR1.1	N/A
UM#38	N/A					N/A
UM#39	VEEPRYPQFP	VEEPRY	6	MACROD1	Q9BQ69.2	N/A
UM#40	NKDDKNVHNAD	NVH+AD	5.5	MEP1A	Q16819.2	N/A
UM#41	YSENSFEQHLD	YSENSF	6	DNAH10	Q8IVF4.4	N/A
UM#42	DWANGHDCPQ	ANG+DC	5.5	ZFYVE9	O95405.2	N/A
UM#43	QCEPDNPDDFL	NPDDFL	6.5	TRIP12	Q14669.1	N/A
UM#44	KFMDKPDTPD	FMDKP+	5.5	ICE2	Q659A1.2	N/A
UM#45	HPWLWFKHT	HPWLW	5	CLGN	O14967.1	N/A
UM#46	CVPWIMGG	PWIMGG	6	RBAK	Q9NYW8.1	N/A

UM#47	ADEWNKVNEFF	KVN+FF	5.5	ITPR2	Q14571.2	N/A
UM#48	RRALSHFPDI	ALSHFP	6	KPNA4	O00629.1	N/A
UM#49	TDREGPDNPIG	REGP+N	5.5	ASPSCR1	Q9BZE9.1	N/A
UM#50	PYMWQPGIG	PYMW+P	5.5	RBSN	Q9H1K0.2	N/A
UM#51	AAFTHDIEK	AAFTHD	6	NID2	Q14112.3	N/A
UM#52	QQGRVPGPGAT	QGR+PGPG	7.5	SIT1	Q9Y3P8.1	N/A
UM#53	WWELTIIEN	ELTII+N	6.5	CACNA1I	Q9P0X4.1	N/A
UM#54	DDVAQRRIPGD	QRRIPG	6	SIN3A	Q96ST3.2	N/A
UM#55	ESMAPNKDKDY	+APNKDK	6.5	FER1L5	A0AVI2.3	N/A
UM#56	AMNQGMFQI	QGMFQ	5	SLC25A35	Q3KQZ1.1	N/A
UM#57	IRLKEFTDYL	KEFTDYL	7	SOS1	Q07889.1	Increased expression in neuronal cells of AD patients [4]
UM#58	DNDPSTYML	D+DPST	5.5	NCKAP5	O14513.2	N/A
UM#59	CKRFDCPTPI	KRF+CP	5.5	SP4	q02446.2	Increased expression in the brain of AD patients; associated with NFT and neuronal apoptosis [15]
UM#60	LNGHPQHSLC	L+GHPQ	5.5	CSMD3	Q7Z407.3	N/A
UM#61	SLRYYNHDIP	LRYY+H	5.5	SBF1	O95248.3	N/A
UM#62	DNIHFLMY	+NIH 리	5.5	CRYBG3	Q68DQ2.3	N/A
UM#63	HMMYPGL	MM+PGL	5.5	DBX1	A6NMT0.2	N/A
UM#64	WMTDEPSGKKPM	+EPSGKK	6.5	MICALL2	Q8IY33.1	N/A
DG#1	TRSEERGNSAN	RSEERG	6	SPEG	Q15772.4	N/A
DG#2	FFEYDQIIRAN	+YDQII	5.5	GDAP1L1	Q96MZ0.2	N/A
DG#3	AEETRMSSRD	TRMSSR	6	JCAD	Q9P266.3	N/A
DG#4	GPWTFTLT	GPWTFTL	7	BPIFB1	Q8TDL5.1	N/A
DG#5	VWNAWDKMK	WDKMK	5	MMAA	Q8IVH4.1	N/A
DG#6	WTNHFEEGVA	HFEEG+	5.5	FREM1	Q5H8C1.3	N/A
DM#1	NWSWRYFV	WRYFV	5	HSMCR30	P0CW71.1	N/A
DM#2	FFETDGKKKRNK	FETDGKK	7	CRTAM	O95727.2	N/A
DM#3	LYQLQWWRFQ	LQWWR	6	POLG2	Q9UHN1.1	N/A
DM#4	TKKEOWOEOKF	TKKE	4	TTN	Q8WZ42.4	N/A
DM#5	NPMFQNNNFF	FQNNN	5	DNAH6	Q9C0G6.3	N/A
DM#6	LNRWYSKEIT	YSKEIT	6	CFAP47	Q6ZTR5.4	N/A
DM#7	FVHYKQYNI	+KQYNI	5.5	PAPPA	Q13219.3	N/A
DM#8	WVNWTHHQTS	WTHHQT	6	CHD1	Q14646.2	N/A
DM#9	AFFLIVY	FFLIVY	6	OR13G1	Q8nGZ3.1	N/A
DM#10	FTWIVTEPA	TWIVT+	5.5	MS4A10	Q96PG2.3	N/A
DM#11	CSWFEKNTK	FEKNTK	6	NINL	Q9Y2I6.2	N/A
DM#12	HNMDECEEE	DECEEE	6	IFT46	Q9NQC8.1	N/A
DM#13	MYPINHTTY	YPINHT	6	MMP23A	O75900.2	N/A
DM#14	QAYQRKNRYVP	KNRYV	5	PTPRC	P08575.2	N/A
DM#15	ERSAEIMCKSV	AEIMCK	6	RPS6KA4	O75676.1	N/A
DM#16	PMRSWDAAQ	SW+AAQ	5.5	ECM1	Q16610.2	N/A
DM#17	IIQIMCQTF	I+CQTF	5.5	DNAH6	Q9C0G6.3	N/A
DM#18	KLITNFNIFQ	LITNFN	6	IPO11	Q9UI26.1	N/A
DM#19	YATDEYLET	TDEYLE	6	SMPD4	Q9NXE4.2	N/A
DM#20	MYKKKKMFSG	YKKKKM	6	CCDC59	Q9P031.2	N/A
DM#21	IYSCFRWKIF	+YSCFR	5.5	GNPAT	O15228.1	Decreased expression in AD mouse and human brain [16]
DM#22	ALNMATRTR	LN+ATRT	6.5	CTLA4	P16410.3	N/A
DM#23	QFQFPWMNY	PWM+Y	4.5	CNTN2	Q02246.1	Decreased in brain of AD patients, correlated with BACE1 [17]
DM#24	KLEYMWYQ	YMWYQ	5	TIGD7	Q6NT04.1	N/A

For peptide ID: UG and UM indicate peptides recognized by upregulated IgG and IgM, respectively, and DG and DM indicate peptides recognized by downregulated IgG and IgM, respectively. “Subject” indicates amino acid sequence(s)

within the target protein that overlap the predicted epitope. “Identity” assigns a value of +1 for consecutive matched amino acids and +0.5 for conserved amino acids.

Abbreviations: AD, Alzheimer’s disease; N/A, not applicable; Aβ, β-amyloid; APP, amyloid precursor protein; SNP, single-nucleotide polymorphism; CA, cornu ammonis; NFT, neurofibrillary tangle.

Supplementary References

1. McKhann GM, Knopman DS, Chertkow H, et al. The diagnosis of dementia due to Alzheimer's disease: recommendations from the National Institute on Aging-Alzheimer's Association workgroups on diagnostic guidelines for Alzheimer's disease. Alzheimers Dement. 2011 May;7(3):263-9.
2. Jespersen MC, Peters B, Nielsen M, Marcatili P. BepiPred-2.0: improving sequence-based B-cell epitope prediction using conformational epitopes. Nucleic Acids Res. 2017 May 02.
3. Baruch K, Deczkowska A, Rosenzweig N, et al. PD-1 immune checkpoint blockade reduces pathology and improves memory in mouse models of Alzheimer's disease. Nat Med. 2016 Feb;22(2):135-7.
4. McShea A, Zelasko DA, Gerst JL, Smith MA. Signal transduction abnormalities in Alzheimer's disease: evidence of a pathogenic stimuli. Brain Res. 1999 Jan 9;815(2):237-42.
5. Tiwari SS, Mizuno K, Ghosh A, et al. Alzheimer-related decrease in CYFIP2 links amyloid production to tau hyperphosphorylation and memory loss. Brain. 2016 Oct;139(Pt 10):2751-65.
6. Reitz C, Tosto G, Vardarajan B, et al. Independent and epistatic effects of variants in VPS10-d receptors on Alzheimer disease risk and processing of the amyloid precursor protein (APP). Transl Psychiatry. 2013 May 14;3:e256.
7. Long J, Pan G, Ifeachor E, Belshaw R, Li X. Discovery of Novel Biomarkers for Alzheimer's Disease from Blood. Dis Markers. 2016;2016:4250480.
8. Nikolaev A, McLaughlin T, O'Leary DD, Tessier-Lavigne M. APP binds DR6 to trigger axon pruning and neuron death via distinct caspases. Nature. 2009 Feb 19;457(7232):981-9.
9. Jung CG, Uhm KO, Miura Y, et al. Beta-amyloid increases the expression level of ATBF1 responsible for death in cultured cortical neurons. Mol Neurodegener. 2011 Jul 5;6:47.
10. Afanador L, Roltsch EA, Holcomb L, et al. The Ca2+ sensor S100A1 modulates neuroinflammation, histopathology and Akt activity in the PSAPP Alzheimer's disease mouse model. Cell Calcium. 2014 Aug;56(2):68-80.
11. Schrotter A, Oberhaus A, Kolbe K, et al. LMD proteomics provides evidence for hippocampus field-specific motor protein abundance changes with relevance to Alzheimer's disease. Biochim Biophys Acta. 2017 Jun;1865(6):703-14.
12. Shang Z, Lv H, Zhang M, et al. Genome-wide haplotype association study identify TNFRSF1A, CASP7, LRP1B, CDH1 and TG genes associated with Alzheimer's disease in Caribbean Hispanic individuals. Oncotarget. 2015 Dec 15;6(40):42504-14.
13. Chibnik LB, White CC, Mukherjee S, et al. Susceptibility to neurofibrillary tangles: role of the PTPRD locus and limited pleiotropy with other neuropathologies. Mol Psychiatry. 2017 Mar 21.
14. Deters KD, Nho K, Risacher SL, et al. Genome-wide association study of language performance in Alzheimer's disease. Brain Lang. 2017 Sep;172:22-9.
15. Boutillier S, Lannes B, Buee L, et al. Sp3 and sp4 transcription factor levels are increased in brains of patients with Alzheimer's disease. Neurodegener Dis. 2007;4(6):413-23.
16. Hossain MS, Abe Y, Ali F, et al. Reduction of Ether-Type Glycerophospholipids, Plasmalogens, by NF-kappaB Signal Leading to Microglial Activation. J Neurosci. 2017 Apr 12;37(15):4074-92.

17. Gautam V, D'Avanzo C, Hebisch M, Kovacs DM, Kim DY. BACE1 activity regulates cell surface contactin-2 levels. Mol Neurodegener. 2014 Jan 9;9:4.
